# Maternal Lineages from 10–11th Century Commoner Cemeteries of the Carpathian Basin

**DOI:** 10.3390/genes12030460

**Published:** 2021-03-23

**Authors:** Kitti Maár, Gergely I. B. Varga, Bence Kovács, Oszkár Schütz, Zoltán Maróti, Tibor Kalmár, Emil Nyerki, István Nagy, Dóra Latinovics, Balázs Tihanyi, Antónia Marcsik, György Pálfi, Zsolt Bernert, Zsolt Gallina, Sándor Varga, László Költő, István Raskó, Tibor Török, Endre Neparáczki

**Affiliations:** 1 Department of Genetics, University of Szeged, H-6726 Szeged, Hungary; kitti.maar@gmail.com (K.M.); schutzoszi@gmail.com (O.S.); endre.neparaczki@bio.u-szeged.hu (E.N.); 2 Department of Archaeogenetics, Institute of Hungarian Research, H-1014 Budapest, Hungary; varga.gergely@mki.gov.hu (G.I.B.V.); kovacs.bence.gabor@mki.gov.hu (B.K.); zmaroti@gmail.com (Z.M.); nyerki.emil@mki.gov.hu (E.N.); tihanyi.balazs@mki.gov.hu (B.T.); 3 Department of Pediatrics and Pediatric Health Center, University of Szeged, H-6725 Szeged, Hungary; kalmar.tibor@med.u-szeged.hu; 4 SeqOmics Biotechnology Ltd., H-6782 Mórahalom, Hungary; nagyi@seqomics.hu (I.N.); latinovicsd@seqomics.hu (D.L.); 5 Institute of Biochemistry, Biological Research Centre, H-6726 Szeged, Hungary; 6 Department of Biological Anthropology, University of Szeged, H-6726 Szeged, Hungary; antonia.marcsik@gmail.com (A.M.); palfigy@bio.u-szeged.hu (G.P.); 7 Department of Anthropology, Hungarian Natural History Museum, H-1083 Budapest, Hungary; bernert.zsolt@nhmus.hu; 8 Ásatárs Ltd., H-6000 Kecskemét, Hungary; gallinazsolt@gmail.com; 9 Department of Archaeology, Institute of Hungarian Research, H-1014 Budapest, Hungary; 10 Ferenc Móra Museum, H-6720 Szeged, Hungary; varga.sandor.arch@gmail.com; 11 Rippl-Rónai Municipal Museum with Country Scope, H-7400 Kaposvár, Hungary; koltolaszlo48@gmail.com; 12 Institute of Genetics, Biological Research Centre, H-6726 Szeged, Hungary; rasko@brc.hu

**Keywords:** ancient mitogenome, Hungarian commoners, Carpathian Basin

## Abstract

Nomadic groups of conquering Hungarians played a predominant role in Hungarian prehistory, but genetic data are available only from the immigrant elite strata. Most of the 10–11th century remains in the Carpathian Basin belong to common people, whose origin and relation to the immigrant elite have been widely debated. Mitogenome sequences were obtained from 202 individuals with next generation sequencing combined with hybridization capture. Median joining networks were used for phylogenetic analysis. The commoner population was compared to 87 ancient Eurasian populations with sequence-based (Fst) and haplogroup-based population genetic methods. The haplogroup composition of the commoner population markedly differs from that of the elite, and, in contrast to the elite, commoners cluster with European populations. Alongside this, detectable sub-haplogroup sharing indicates admixture between the elite and the commoners. The majority of the 10–11th century commoners most likely represent local populations of the Carpathian Basin, which admixed with the eastern immigrant groups (which included conquering Hungarians).

## 1. Introduction

Hungarian history was profoundly determined by the conquering Hungarians (succinctly, the Conquerors), who arrived at the Carpathian Basin from the Eastern European steppe at the end of the 9th century AD as an alliance of seven tribes. The leaders of the alliance (Álmos and his son Árpád) founded a steppe state upon the ashes of the Avar Khaganate [1,2], and their descendants later established the Hungarian Kingdom. The archaeological legacy of the Conquerors is well defined, especially in the small 10th century cemeteries of the military leader strata whose grave finds included precious metal jewels and costume ornaments as well as decorated horse riding- and weapon-related grave goods [3]. Most of the larger cemeteries attributed to the common people are dated somewhat later, to the 10–12th centuries. People in these so-called village cemeteries were buried with simpler jewels and grave goods, with the sporadic appearance of weapons or harness accessories. There is a general agreement that elite graves with typical grave goods represent first- or second-generation immigrant Conquerors, but the affiliations of people in the village cemeteries are far less clear. For 50 years, they were identified with the Bijelo Brdo culture of the local Slavic people, until their relation to the Conquerors was recognized in 1962 [4] (see Appendix A for details), but to what extent they can be identified with the immigrants as opposed to the previous local population is not yet clear. The answer to this question considerably determines the historical interpretation of the conquest and subsequent events in the Carpathian Basin, and genetic data may contribute to clarifying this issue.

Hitherto, most genetic studies were focused on the elite graves, as these promised an answer for the origin of the immigrant groups. In [5], 76 individuals were selected from 23 cemeteries mainly representing the 10th century elite, and 23% of the maternal lineages identified from hypervariable region (HVR) sequences were east Eurasian and 77% were west Eurasian. Another study, [6], aimed at characterizing the population of entire group of elite cemeteries, sequencing 102 mitogenomes (30% of which had Central–Inner Asian maternal ancestry, while most of the remaining lineages originated from western Eurasia). Y-chromosome studies [7] found that male lineages had similar phylogeographic compositions to female ones. Thus, all studies had congruent results, inferring that the Conqueror elite population originated from an admixture of Asian and European groups on the Pontic steppe.

This raises the question of whether the commoners were genetically similar to the elite, and, if so, could they be one and the same population, or did the poorer strata have a different origin? This question was addressed in the first HVR-based study [8], in which 27 selected graves from 15 cemeteries were grouped according to the type of grave goods present, and the population with “classical” grave goods were found to contain a higher proportion of east Eurasian haplogroups (Hgs) than the group with poor archaeological remains. However, this conclusion was based on a small sample size and a low resolution HVR study, and a systematic characterization of the commoner population with a representative dataset has not been performed yet.

We set out to implement a comprehensive study in this matter, and to this end, we selected eight cemeteries archaeologically evaluated as belonging to the 10–11th century commoners, from which we obtained 202 whole mitogenome sequences. Phylogenetic analysis was performed to illuminate the origin of each maternal sub-Hg of the studied remains. We compared the mitochondrial haplogroup compositions of the commoner and elite populations to find out their genetic relationship and applied different population genetic methods to elucidate the relationship of the commoners with other ancient Eurasian populations. For this reason, we also built a comprehensive database of ancient Eurasian populations, which included all available published mitogenome data.

## 2. Materials and Methods

### 2.1. Archeological Background

**Figure 1:**
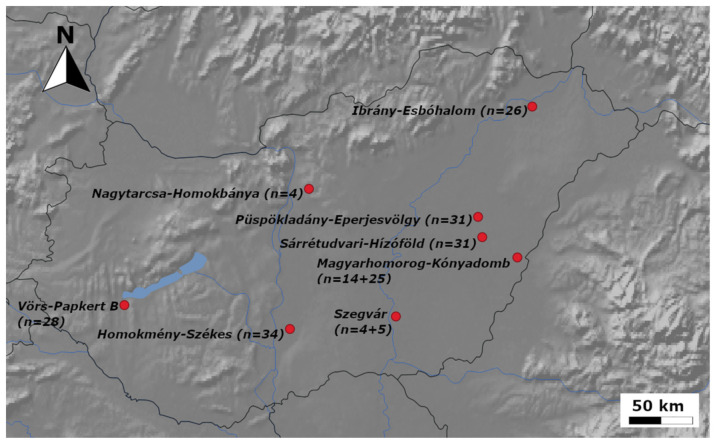
The locations of the graveyards of the Hungarian commoners (ConqC) under study. Sample size is indicated next to cemetery names; two numbers in Magyarhomorog and Szegvár indicate that two nearby cemeteries were sampled. The map was generated using QGIS 3.12.0 [10].

In contrast to the small 10th century cemeteries with characteristic grave goods [9] representing the conquering Hungarian elite (ConqE), archaeologists classify large 10–11th century cemeteries containing poor grave goods with the sporadic appearance of ConqE findings (see Appendix A for details) as belonging to Hungarian commoners (ConqC). We collected petrous bones (or where these were unavailable, teeth) from 229 human remains from 10 archeological sites (Figure 1) associated with Hungarian commoners.

We made an effort to carry out representative sampling. Thus, graves were selected from each section of the cemeteries (including males and females from burials both with and without grave goods and all anthropological types). The number of collected, processed and analyzed samples from each cemetery is summarized in Table 1.

The largest 10th century commoner cemetery with 262 graves was excavated in Sárrétudvari-Hízóföld [11] (Appendix A.2.6), with a high proportion of graves containing archery equipment and stirrups. We recovered 31 mitogenomes from this site, and a further 8 sequences were added from our previous study [6,12].

Another large commoner cemetery with 637 graves is located in the nearby Püspökladány-Eperjesvölgy [11] (Appendix A.2.5). This cemetery contains a “pagan” and a “Christian” section. Both sections of the graveyard were sampled and we obtained 31 mitogenomes.

The Ibrány-Esbóhalom commoner cemetery with 269 graves also stretches into the Christian era [13] (Appendix A.2.2). We analyzed 32 remains from this site, resulting in the obtainment of 26 mitogenomes.

We studied 36 remains from the Homokmégy-Székes cemetery excavated at the Duna-Tisza Interfluve [14] with 206 graves, which was referred to by the archaeologist as a “typical cemetery of conquest period commoners” (Appendix A.2.1), and obtained 34 mitogenomes.

Among the studied cemeteries, Magyarhomorog-Kónyadomb [15] (Appendix A.2.3) is an exceptional case, as archaeologically it can be divided into two sections: a small section with 17 individuals was used by the 10th century Conqueror elite, while the larger section with 523 graves of 10–11th century commoners raises the question of potential continuity. We sequenced 14 samples from the elite section and 25 samples from the commoner section.

From the Transdanubia region, we included the Vörs-Papkert-B cemetery [16] (Appendix A.2.9), the 716 excavated burials of which are mostly from the late Avar and Carolingian periods. However, 33 people can be dated to the time of the Hungarian conquest. The uninterrupted usage of this graveyard raises the possibility that it might represent the same population in the subsequent periods; thus, we sampled graves from each period as indicated in Table 1.

Finally, we complemented our sample set with a few individuals from the Nagytarcsa-Homokbánya (Appendix A.2.4), Szegvár-Oromdűlő (Appendix A.2.7) and Szegvár-Szőlőkalja (Appendix A.2.8) commoner cemeteries, as listed in Table 1. All of the 13 samples came from poor burials or from graves devoid of archaeological grave goods. For a detailed description of the sites and archaeological findings, see Table S1.

### 2.2. Library Preparation, Sequencing and Hg Assignment

All pre-PCR laboratory procedures leading to next generation sequencing (NGS) were conducted in the common ancient DNA laboratory of the Department of Archaeogenetics of the Institute of Hungarian Research and Department of Genetics, University of Szeged, Hungary. Details concerning the ancient DNA purification, library preparation, hybridization capture, sequencing and sequence analysis method are given in [12]. We used the double stranded library protocol of [17] with double indexing [18]. All libraries were made from partial uracil-DNA glycosylase (UDG)-treated DNA extracts [19]. We estimated the endogenous human DNA content of each library with low coverage shotgun sequencing (Table S2a). Then, the mitogenomes from samples with similar proportions of human DNA content were pooled and enriched together according to [20]. Captured and amplified libraries were purified on MinElute columns. Quantity and quality measurements were performed with the Qubit fluorometric quantification system and the TapeStation automated electrophoresis system (Agilent). A further 13 mitogenome sequences were obtained from whole genome sequencing, as indicated in Table 1 and Table S2.

The adapters of paired-end reads were trimmed with the Cutadapt software [21] in paired end mode. Read quality was assessed with FastQC [22]. Sequences shorter than 25 nucleotides were removed from this dataset. The resulting analysis-ready reads were mapped to the GRCh37.75 human genome reference sequence that also contains the mtDNA revised Cambridge Reference Sequence (rCRS, NC_012920.1) [23] using the Burrows Wheeler Aligner (BWA) v0.7.9 software [24] with the BWA mem algorithm in paired mode and default parameters. Samtools v1.1 [25] was used for sorting and indexing binary alignment map (BAM) files. PCR duplicates were removed using Picard Tools v 1.113 [26]. Ancient DNA damage patterns were assessed using MapDamage 2.0 [27] and read quality scores were modified with the rescale option to account for post-mortem damage. Mitochondrial genome contamination was estimated using the Schmutzi algorithm [28] (Table S2b). Mitochondrial haplogroup (Hg) determination was performed using HaploGrep v2.1.25 [29] (Table S3a). The biological sex of the individuals was identified according to [30] based on the X/Y ratio of the reads obtained from shotgun sequencing.

The raw nucleotide sequence data of the 202 samples were deposited to the European Nucleotide Archive (http://www.ebi.ac.uk/ena) under the accession number: PRJEB40566.

### 2.3. Assembling an Ancient Eurasian Mitogenome Database

For the phylogenetic and population genetic analyses, we built a database containing 4191 published ancient Eurasian mitogenomes (Table S4). Sequences were downloaded from the NCBI and the European Nucleotide Archive databases. Where it was necessary, mitogenome sequences were sorted out from whole genomes. This database was then augmented with the 202 new mitogenomes from this study. We ordered the published samples into 88 populations based on time range, archaeological site and context, as well as the classification of the published genome data. In cases when populations were under-represented due to a low sample size, we grouped samples from related cultures like Alans and Saltovo-Mayaki, Medieval samples from Italy, Germany and England, Iberian Chalcolithic and Bronze Age samples, Chalcolithic samples from Iran and Turan, early and late Sarmatians, etc. (Table S4).

### 2.4. Phylogenetic and Population Genetic Study

A sub-set of the published sequences was of poor quality. We excluded sequences with >5% missing data from the phylogenetic and Fst analysis and used 3844 fasta files of ancient sequences and 11,682 fasta files of modern sequences for building median joining (MJ) networks, as described in [6]. The phylo-geographic origins of the samples were assessed from the geographic origin of the nearest Hgs. We distinguished four regions: east Eurasia, west Eurasia, Eurasia and Caucasus–Middle East (Figure S1).

For population genetic analysis, we merged all 169 ConqC data to a single population (Tables S3c and S4), excluding members of the elite Magyarhomorog cemetery as well as Avar and Caroling samples from the Vörs-Papkert cemetery (excluded samples are color labeled in Table S2b). These were supplemented with 13 commoner mitogenomes published previously [6], as listed in Table 1. The merged ConqC population was compared to the 88 ancient Eurasian groups from the newly assembled mitogenome database, including the previously published military elite strata of the Conquerors [6,12,31], which was supplemented with the Magyarhomorog elite graveyard data from the present study (Table 1, Tables S3c and S4).

Three independent methods were applied to measure the genetic distances of ConqC from other ancient populations. In the first analysis, we reduced the Hg assignments of all samples to major Hgs, which decreased population data to 34 dimensions, which is sufficient to display the main correlations. Then, major Hg frequencies were calculated and principal component analysis (PCA) was conducted, employing the function “prcomp” in R 3.6.3. [32]. We also compared the major Hg frequencies of the ConqC and ConqE groups separately.

In a second approach, a traditional sequence-based method was implemented, calculating pair-wise population differentiation values (Fst) with Arlequin 3.5.2.2 [33] from whole mtDNA genomes, as described in [6]. Multi-dimensional scaling (MDS) was applied on the matrix of linearized Slatkin Fst values [34], and the values were visualized in the two-dimensional space using the cmdscale function implemented in R 3.6.3 [32].

In a third approach, shared haplogroup distance (SHD) values were measured between the populations according to our previous study [6], which calculates the frequency of identical terminal sub-Hgs (the deepest determined Hg level) between populations, as these testify shared ancestry or past admixture.

## 3. Results

### 3.1. Sequencing Results and Assigned Haplogroups

We collected a total of 229 samples from the listed sites, but could not obtain DNA from 13 samples. Another 10 samples were excluded from the analysis due to low mitogenome sequence coverage and 3 further samples were excluded due to high contamination values. Using the NGS method combined with target enrichment, we acquired 189 ancient mitogenome sequences, and a further 13 were obtained from whole genome sequencing; thus, we report 202 new mitogenomes in this paper (Table S3a). We obtained 4.2-3068x mitogenome coverage, and the average coverage was 231x. Schmutzi estimated negligible contamination for most of the 202 samples. Seven samples were indicated to carry significant (15–21%) contamination; nonetheless, Schmutzi could determine the endogenous sequence unambiguously for these samples due to high coverage, enabling a correct Hg assignment. For details of the sequencing data, see Table S2. On the grounds of haplogroup determination by HaploGrep 2.0, the 202 samples belong to 154 sub-Hgs and 187 different haplotypes (Table S3a).

### 3.2. Kinship Analysis

We examined a possible kinship relation between and within cemeteries. We detected 10 pairs of identical mitochondrial haplotypes within cemeteries and 4 pairs between cemeteries (Table S3b), which indicate a potential direct maternal relationship of these individuals, but this of course is not inherent evidence of close family relations.

### 3.3. Phylogenetic Analysis

**Figure 2:**
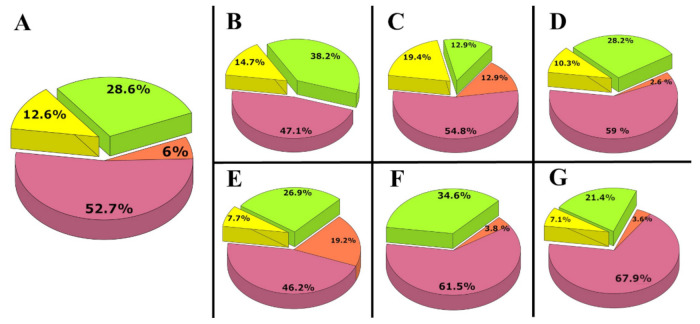
The phylogeographic origin of the ConqC maternal lineages from different cemeteries. Data are summarized from Figure S1 and from a previous study [6]. West Eurasian haplogroups (Hgs) are marked with pink, east Eurasian Hgs are marked with yellow, Eurasian Hgs are marked with green and Caucasus–Middle East Hgs are marked with brown. (**A**) Distribution of the merged data of 182 Hungarian commoner samples from all cemeteries. (**B**–**G**) The phylogeographic distribution of the maternal lineages from individual cemeteries: (**B**) Homokmégy-Székes (n = 34); (**C**) Püspökladány-Eperjesvölgy (n = 31); (**D**) Sárrétudvari-Hízóföld (n = 39); (**E**) Ibrány-Esbóhalom (n = 26); (**F**) Magyarhomorog-Kónyadomb (n = 26, with samples taken just from the commoner part); (**G**) Vörs-Papkert-B (n = 28, including all samples from this cemetery).

As some of the mitochondrial sub-clades may have specific geographical distribution [35,36], we elucidated the phylogenetic relations of each mitogenome sequence using M–J networks, as shown in Figure S1. The closest sequence matches pointed at a well-defined geographical region in most cases, which is indicated next to the phylogenetic trees and is summarized on Figure 2.

Phylogenetic trees revealed that, out of the 182 commoner maternal lineages, 23 were unequivocally derived from east Eurasia and 107 were derived from west Eurasia, while 52 are widespread throughout Eurasia. Out of the western Eurasian lineages, 11 have a primarily Caucasus–Middle East distribution (Figure 2A).

### 3.4. Haplogroup Composition of Individual Cemeteries

The 34 investigated samples from Homokmégy-Székes belonged to 30 Hgs (Table S3a). As for the lineages, 47.1% were of European origin and 14.7% were of east Eurasian origin, while 38.2% showed general Eurasian distribution (Figure 2B).

From the Püspökladány-Eperjesvölgy cemetery, 31 remains were analyzed. The maternal lineages were classified into 28 Hgs (Table S3a), and they showed 54.8% west Eurasian ancestry, 19.4% east Eurasian ancestry and 12.9% Eurasian ancestry, while 12.9% had a Caucasus–Middle East affinity (Figure 2C).

The newly reported mitogenomes of 31 individuals from Sárrétudvari-Hízóföld belonged to 26 Hgs (Table S3a). In a previous study, the mitochondrial lineage of eight individuals from this cemetery were obtained [6]. Merging these data, 59% of the lineages had west Eurasian ancestry, 10.3% had east Eurasian ancestry, 28.2% had Eurasian ancestry and 2.6% had Caucasian–Middle Eastern maternal ancestry (Figure 2D).

The Ibrány-Esbóhalom cemetery was represented by 26 samples falling to 26 different Hgs (Table S3a). 46.2% of the maternal lineages originated from Europe, 7.7% originated from east Eurasia and 19.2% originated from the Caucasus–Middle East region, while 26.9% of the lineages had a Eurasian distribution (Figure 2E).

We sequenced 14 mitogenomes out of the 17 remains from the elite part of Magyarhomorog-Kónyadomb, and their Hg composition was very similar to those of previously studied elite cemeteries [6]; 35.7% of the lineages were of east Eurasian origin, 42.9% were of European origin and 21.4% were of Eurasian origin (Table S1). The high frequency of N1a1a1a1a and T1a1, as well as the occurence of N1a1a1a1 and D4 in this small cemetery, finds its best parallels in the Karos and Kenézlő elite graveyards [4], supporting the archaeological evaluation; thus, we included these data in the elite dataset (Table S3c).

From the 11–12th century commoner part of Magyarhomorog, we sequenced 25 samples which belonged to 22 mitochondrial Hgs (Table S3a), supplemented with one published sample from this site [6]. From the 26 samples, 61.5% had a west Eurasian origin, 34.6% had an Eurasian origin and 3.8% had a Caucasus–Middle East affinity (Figure 2F); thus, genetic data also corroborated the hypothesis that the large graveyard represents a separable commoner population.

The cemetery of Vörs-Papkert is another special case, as it was used for centuries by successive populations of Avars, Carolingians and Conquerors populations. Evaluating the entire 28 sample set from this cemetery together (Figure 2G) showed a very similar overall picture to that of other commoner cemeteries, with 25 Hgs, 67.9% of which had a west Eurasian origin, 7.1% had an east Eurasian origin, 21.4% had an Eurasian origin and 3.6% had a Caucasus–Middle East affinity. Hg H dominated this graveyard, as 16 out of the 28 remains belonged to Hg H irrespective of historical period. A single D4e4 Hg was detected among the studied ConqC and a single A16 was detected among the Avar period samples as weak signs of Asian impact (Table S1). By all means, for the population genetic analysis, we removed Avar and Carolingian samples from this dataset.

The six ConqC graveyards with a meaningful sample size showed a rather similar overall picture, with an average of 12.6% east Eurasian Hgs almost confined to C and D, which allowed us to infer a similar overall east Eurasian impact throughout cis-Danubia.

We also investigated a few individuals from other commoner cemeteries, namely four samples from Nagytarcsa-Homokbánya, four samples from Szegvár-Oromdűlő and five samples from Szegvár-Szőlőkalja, resulting in two east Eurasian lineages besides the European ones (Table S3a).

We acknowledge that the average of 30 samples per site may poorly represent the individual cemeteries, but the total number of 182 commoner remains (Table S3c) can be regarded as considerably representative for population genetic analysis.

### 3.5. Population Genetic Analysis

**Figure 3:**
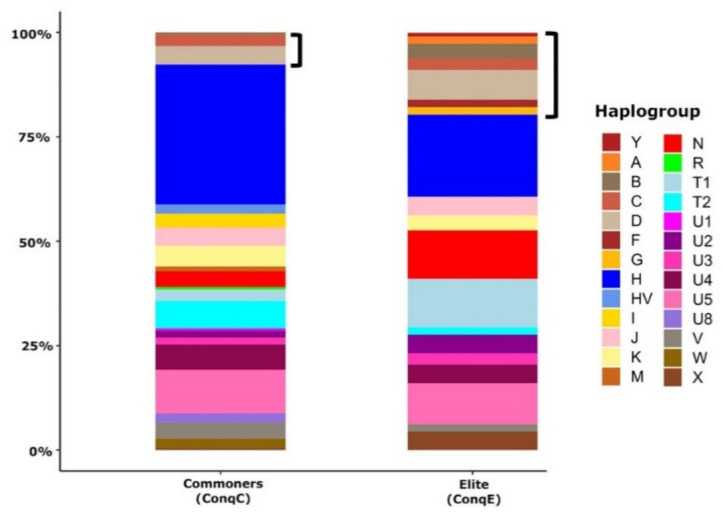
Comparison of the major Hg distributions from ancient Hungarian populations. The major Hg distribution of commoner samples (n = 182) from this study is distinct from that of Conqueror elite samples (n = 112) taken from previous studies [6,12,31], including elite data from Magyarhomorog in the present study. Brackets mark east Eurasian Hgs.

First, we compared the major Hg distribution of the conqueror period elite and commoner populations (Figure 3). The heterogeneity of the major Hg distribution of ConqE is comparable to that of ConqC (22 and 19 main Hgs, respectively); however, the Hg compositions of the two groups show considerable differences. The ratio of east Eurasian major-Hgs in the commoners is 7.69%, contrary to the 19.64% of the elite. The elite contains a broad spectrum of east Eurasian Hgs (A, B, C, D, F, G and Y), while only C and D occur with notable frequency in the commoners, with a single appearance of B.

West Eurasian Hgs of ConqC and ConqE also show notable differences: Hgs HV, I, M, R, U1, U8 and W occur with moderate frequencies in commoners, while these are completely absent from the elite population. Three Hgs (N, T1 and X), typically widespread both in east and west Eurasia, show much higher ratio in the elite than in commoners: N’s ratios are 11.61% in the elite population and 3.85% in the commoner population; T1’s ratios are 11.61% in the elite population and 2.75% in the commoner population; and X’s ratios are 4.46% in the elite population and 0.55% in the commoner population. The opposite is true for Hgs H and T2; among commoners, H is the most prevalent Hg with a 33.52% frequency, while in the elite group, its proportion is significantly lower (19.64%); T2 has a 6.59% proportion in the commoner population and a 1.79% proportion in the elite population.

**Figure 4:**
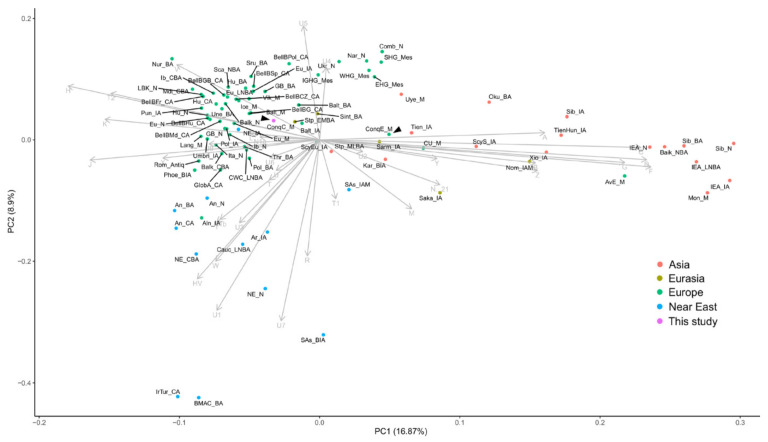
The principal component analysis (PCA) plot of the major mtDNA haplogroup distribution (distinguishing Hgs A, B, C, D, F, G, H, HV, I, J, K, L, M, N, N1a, N1b, R, T, T1, T2, U, U1, U2, U3, U4, U5, U6, U7, U8, V, W, X, Y and Z) of 88 Eurasian populations. The abbreviations of the population names are given in Table S4b. Color shadings denote geographic regions as indicated. ConqC and ConqE are highlighted with arrowheads. PC1 separates European populations to the left and Asian populations to the right side. PC2 separates Anatolian–Caucasus groups to the bottom and hunter–gatherers to the top.

As the Hg composition of the studied commoner samples markedly differs from that of the elite, we measured ConqC’s genetic distances from ConqE as well as its distances from 87 published ancient Eurasian populations (Table S5). PCA obtained from the major Hg frequencies of 88 populations (Figure 4) highlights the distance between ConqE and ConqC. The ConqC clustered in the eastern side of the European aggregation, with the closest genetic affinity to Baltic Bronze Age populations, Baltic Iron Age populations, Baltic Medieval populations, Bell Baker Germany and Great Britain Bronze Age populations, and is not far away from the Steppe Early-Middle Bronze Age (Steppe EMBA) population, though these relative distances need to be interpreted with care, as our population dataset certainly incompletely represents the past genetic variability. In contrast, the Conqueror Elite is located between ancient European and Asian populations and its closest clusters are the Sarmatian Iron Age population, the Tien Shan Iron Age population, the Karasuk late Bronze Age population and the two groups suggested to be in connection with the Conquerors [31]: the Cis-Ural Medieval population and the Uyelgi Trans-Ural Medieval population.

**Figure 5:**
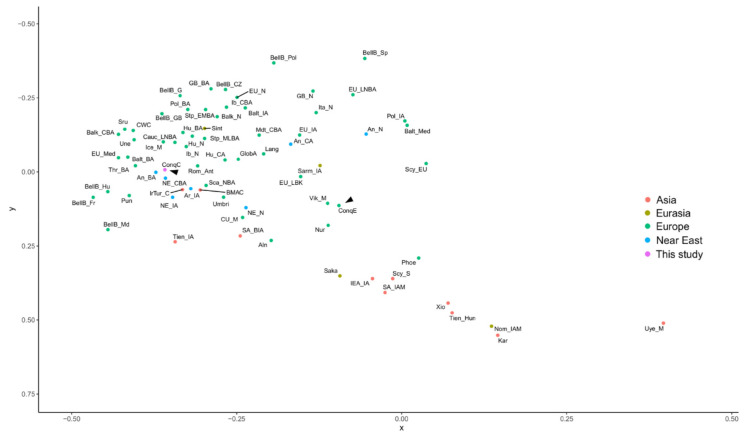
A multi-dimensional scaling (MDS) plot from the linearized Slatkin Fst values from Table S5a. Abbreviations of the population names are given in Table S4b. European populations are sequestered to the left and Asian populations are sequestered to the bottom right. Color shading denotes geographic regions as indicated. ConqC and ConqE are highlighted with arrowheads.

In order to further reveal the genetic relationships of ConqC with other ancient groups, we drew an MDS plot (Figure 5) from linearized Slatkin Fst values (Table S5a). Fst distances confirmed that ConqC is nearest to ancient European and Near Eastern populations; in the Pairwise Fst matrix, the closest groups are the European Medieval (0.0098), Anatolia Bronze Age (0.00991), Iceland Medieval (0.01433), pre-Roman (Umbri) Iron Age from Italy (0.01691) and Roman Antiquity (0.01701) groups, followed by other European Bronze Age, Neolithic and Chalcolithic groups. Accordingly these are located close on the MDS plot. On the other hand, Fst data show that ConqC significantly differs from ConqE (*p* < 0.00000); in other words, the probability that the two populations are identical is below 1/100,000.

The novel SHD population genetic method gave similar results, but also revealed new information (Table S5b). ConqE has the smallest SHD distance from ConqC, followed by European populations from the Neolithic to the Medieval periods. It is also notable that the SHD and Fst distances of Steppe EMBA populations are comparable to those of European groups. European Scythian and Scytho–Siberian populations have noteworthy SHD distances as well, indicating that ConqC significantly shared sub-Hgs with these Eurasian steppe populations.

## 4. Discussion

In this paper, an attempt was made to provide a genetic description of the common people of the Carpathian Basin who lived in the 10–11th centuries during the period of the Hungarian conquest. Of the 202 obtained mitogenomes, 169 belonged to commoners, while 14 samples from the Magyarhomorog cemetery were revealed to represent a small elite graveyard, not related to the adjacent commoner remains. The elite has been shown to comprise around 30% of the east Eurasian Hgs, including characteristic ones like N1a1a1a1a [6] (Table S3c).

The overall Hg composition of the commoner population proved to be significantly different from that of the elite with respect to both east and West Eurasian Hgs, indicating that these two groups likely had different origins. Population genetic analysis definitely clustered ConqC primarily with European and Near Eastern populations, separating them from the elite, suggesting that people with local European origin dominated the ConqC population.

The presence of a non-negligible proportion of east Eurasian Hgs in the ConqC population is a clear sign of admixture with eastern immigrants, presumably with Avars and/or Conquerors. This effect distinguishes ConqC from contemporary European populations, as well as from modern Hungarians, in whom east Eurasian Hgs are negligible. Thus, despite their significant differences, ConqC might have admixed with ConqE to some extent.

This admixture is clearly validated by the SHD method, as ConqC had the smallest SHD distance from ConqE (Table S5b), meaning that out of the studied ancient populations, ConqE shared the highest proportion of identical Hgs with ConqC, best explained by admixture. As the SHD value perfectly represents the common gene pool, the SHD distance of 0.85 indicates a 14% common gene pool between ConqE and ConqC. Out of the 18 shared Hgs, 4 had an east Eurasian origin (Table S3c), so these were very likely transferred from the elite to the commoners. It is especially telling that the most frequent ConqE Hgs (N1a1a1a1, its derivative N1a1a1a1a and T1a1) were present in numerous commoner cemeteries. The east Eurasian N1a1a1a1 ConqE marker most likely originated from the Afanasievo or Sintashta–Tagar cultures [37,38], while, despite its general Eurasian range, a Mongolian Chemurchek–Uyuk–Deer stone–khirigsuur [39] origin of T1a1 in ConqE is very plausible (Table S3c). The close SHD distance of ConqE to Steppe EMBA and Steppe MLBA populations (Table S5b) implies that the Steppe EMBA affinity of ConqC, observed in Figure 4, can also be a consequence of ConqE admixture.

The phylogeographic origin of shared Hgs also signals a possible reciprocal gene flow from ConqC to ConqE, as some of their shared Hgs (H7, K1c1, T2b and V7a) were absent from east Eurasia but had been present in the Carpathian Basin from the Neolithic–Bronze Age, as shown in Table S3c. As a consequence, the 14% common gene pool between ConqE and ConqC cannot be interpreted as a headcount proportion of immigrants and local people. Furthermore, both could have acquired common elements from other unknown populations.

The contemporary local population is descended from previous peoples of the Carpathian Basin, and it has indeed been shown that a large number of people survived to the 10th century from the previous Avar period [40,41]. The Avars also brought along a package of east Eurasian Hgs, and a significant fraction of east Eurasian Hgs which are found in ConqC and are not shared with ConqE (such as B5b4, C4a1b, C5b1a, D4b1, D4e4, D4l2, D4m2a and D5a3a1, as shown in Table S3c). These Hgs are potential candidates for Avar heritage.

## 5. Conclusions

For more accurate conclusions, future investigations are necessary, including high-resolution genome analysis of commoner and elite cemeteries. Additionally, genome data from the pre-Avar, Avar and later Árpádian populations would provide a more complete picture about the exact contribution of subsequent nomadic migrations to the demographic history of the Carpathian Basin.

## Figures and Tables

**Table 1:** Summary of the studied sample size from each cemetery. The mitogenome sequence was obtained after hybridization capture or whole genome sequencing (WGS) as indicated. Samples represent 10–11th century commoners except 14 samples from Magyarhomorog and 20 samples from Vörs-Papkert B. As indicated, we also co-analyzed 13 previously published mitogenomes with new data from this study.

Archaeological Site	Dating (Century CE) Type of Cemetery	No. of Graves	Collected Samples in This Study	Obtained Mitogenomes in This Study (Capture or WGS)	Previously Published Mitogenomes	No. of Samples Analyzed
**Sárrétudvari-Hízóföld**	10th commoner	262	32	31 (capture)	8	39
**Püspökladány-Eperjesvölgy**	10–12th commoner	637	36	31 (capture)		31
**Ibrány-Esbóhalom**	10–11th commoner	269	32	26 (capture)		26
**Homokmégy-Székes**	10–11th commoner	206	36	34 (capture)		34
**Magyarhomorog-Kónyadomb (10–11th century commoner)**	10–11th commoner	523	27	25 (capture)	1	26
**Magyarhomorog-Kónyadomb (10th century elite)**	10th elite	17	14	14 (capture)		14
**Vörs-Papkert B**	8–9th	716	Avar period: 9	8 (capture)		8
	9–10th		Carolingian period: 11	11 (capture)		11
	10–11th commoner		Conquest period: 10	9 (capture)		9
**Nagytarcsa-Homokbánya**	10–11th commoner	21	4	4 (WGS)		4
**Szegvár-Oromdűlő**	10–11th commoner	372	7	4 (WGS)	2	6
**Szegvár-Szőlőkalja**	10th commoner	62	11	5 (WGS)		5
**Orosháza-Görbicstanya**	10th commoner	3			1	1
**Szabadkígyós-Pálliget**	10th commoner	17			1	1

## Data Availability

The data presented in this study are openly available in to the European Nucleotide Archive (http://www.ebi.ac.uk/ena) under the accession number: PRJEB40566.

## Supplementary Materials

The following are available online at https://www.mdpi.com/2073-4425/12/3/460/s1, Figure S1: MJ Networks, Table S1: Summary and archaeological details of studied samples, Table S2a: Shotgun sequence data, Table S2b: Mitogenome sequence data, Table S3a: SNP positions of the mitogenomes, Table S3b: List of identical haplotypes. Table S3c: List of ConqC and ConqE individuals and shared haplogroups Table S4: Ancient mitogenome database, Table S5a: Pairwise Fst matrix of ancient populations, Table S5b: SHD distance matrix of ancient populations.

## Appendix A. Brief Archaeological Background of the Hungarian Commoner Cemeteries

(Detailed archaeological and anthropological description of each graves is provided in Table S1)

### Appendix A.1. A Brief Summary of the Problems Associated with the Archeological Categorization of 10–11th Century Cemeteries in the Carpathian Basin

Assessments of the archeological horizon, cemeteries, and individual burials of the 10–11th century Carpathian Basin, which is the period of the Hungarian conquest and state formation, have undergone significant changes over the past 150 years. The first major summary and categorization of the finds was made by József Hampel [3] based on dating patterns (tombs that could be dated with a coin, tombs that could not be dated with a coin, and stray finds). However, this was soon replaced by his new classification based on ethnicity in which two groups were distinguished: the findings of newly arrived conquerors (the time covering ca. 150 years, 895–1050) buried with horse-riding- and weapon-related grave goods, often with coins of foreign origin (Western European, Byzantine, Arabian), were referred to as Group A and the local population resting in cemeteries composed of rows of graves and buried with simpler jewels and grave goods, often with coins of the kings of the House of Árpád, were referred to as Group B. The latter group later spread in the public consciousness as the Bijelo Brdo culture and was defined as having Slavic ethnicity.

In the first half of the 20th century the number of archeological findings of the 10–11th century period increased rapidly. During the collection and systematization of these finds, Béla Szőke noticed that Group A and Group B cannot be sharply separated, because the findings of the two groups often appear simultaneously in the cemeteries and the cemeteries composed of rows of graves are also present in the central areas of the 10th century Hungarian territory. Based on his observations, he believed that the differences did not indicate different ethnicities, rather they reflected the social status. Thus he developed a new kind of systematization in which the ethnic-based classification was replaced by a division into social categories [4]. Hampel’s Group A was identified with the leading and middle class of the conquerors, and, in connection with Hampel’s Group B, he assumed that they were the findings of the commoner people of the 11th century who were mostly of Hungarian origin but often mixed with other ethnicities (e.g., in the peripheral areas). However, the analysis of the archeological material related to the large-scale excavations of the second half of the 20th century already highlighted the problems of the method and its categories (e.g., [42]); Szőke’s system was used until the last decades to classify individual burials and cemeteries. In addition to the fact that international research has shown that it is not possible, or very difficult, to distinguish legal and social categories using archeological methods alone [43,44], the social categories have not been sufficiently defined, so, in the field of practice, cemeteries were classified in a highly subjective way. On the other hand, the size of the cemetery significantly influenced the evaluation of the findings (e.g., the presence of 3–4 graves with horse-riding- and weapon-related grave goods in a cemetery of 10–20 graves already elevated the given cemetery to the middle class; however, the same ratio in a cemetery composed of 100 graves resulted in the site being classified as a commoner cemetery). However, based on the most recent investigations, only a low percentage of the 10th century cemeteries can be considered as fully excavated, often resulting in erroneous conclusions [42]. In addition, the cemeteries frequently contained a mix of “rich” and “poor” grave goods or even burials without known grave goods, which draws attention to the fact that a cemetery may not necessarily correspond to a particular social category.

István Bóna attempted to reinterpret the 10–11th century cemeteries by distinguishing two types of cemeteries [45]. The so-called military cemeteries were characterized by the rather low number of graves covering one or two generations and often showing a male surplus, which he considered were mostly used in the first half of the 10th century, while the larger village cemeteries with 30–100 graves were used later, during the 10–12th centuries.

This criteria system was further developed by László Kovács, who, breaking with the ethnic and social classification, based the assessment of the sites on the chronological characteristics and quantity of the burials [46]. Within the archeological horizon of the 10–12th centuries, he distinguished two main types in connection with the supposed form of the possibly related settlement: the so-called quarter cemetery and the village cemetery. The quarter group included smaller cemeteries with 5/10–50/75 burials, which were used for a shorter time period and were abandoned between the second half of the 10th century and the turn of the millennium. These cemeteries were characterized by mostly the types of objects and burial customs associated with the conquerors, and at the same time the coins of the kings of the Árpád Dynasty were completely missing. The other main group, the village cemeteries, was characterized by a larger number of graves of 50–200 and above, and their use can be traced back to the 10th, 10–11th, and 10–12th centuries, depending on the sub-type. In connection with the grouping, it may be a problem to separate the 10th century quarter type and the 10th century village type, as there are overlaps based on the time of use and the number of graves, so sometimes the richness and quality of the finds may make the difference [42], but problems with the representational value and symbolic aspects of the objects [47,48] may mislead the conclusions. Questions arise relating to the evaluation of cemeteries dating from the 10th century which were in use through the 11–12th centuries, because based on the written sources concerning the period of the foundation of the Hungarian Kingdom and the early Árpádian age, as well as the results of micro-regional archeological research, significant internal population movements and organized settlements must be taken into account. With the help of archeological methods alone, it is not possible to determine beyond any doubt whether the 10th century and later cemetery parts are contiguous with each other or whether a population change has taken place in the meantime [42]. However, this can also significantly affect the grouping of cemeteries (e.g., a site can be assessed either as a larger 10–11th century village cemetery or as separately having a 10th century quarter/village cemetery and a 11th century village cemetery).

Several methods have been developed since the beginning of research to categorize 10–11/12th century cemeteries and burials. Each method has its advantages and disadvantages depending on the issues raised by the particular research. Being aware of all this, during the short archeological summary of the cemeteries involved in our research, we tried to describe their characteristics objectively; however, in each case, we indicated where the given site was classified in the Szőke and Kovács classifications.

### Appendix A.2. Archeological and Anthropological Description of the Studied Cemeteries

#### Appendix A.2.1. Homokmégy–Székes (Bács–Kiskun County)

The excavation of the site was carried out by Zsolt Gallina and Sándor Varga between 1996 and 2002 [14]. The cemetery, which consisted of 206 graves, is divided into northern and southern parts, based on the type and the orientation of the graves, the grave goods, and the position of the arms. The northern part is further split into east and west sides, based on the density of the graves. Due to the characteristics of the soil, the shape of the graves has been preserved well: several grave pits with sidewall niches have been found, as well as traces of other Avar-age burial customs, which were quite rare in the 10th century (e.g., patterns of post holes in the grave pit). The archeological findings include jewelry and clothing ornaments, such as hoops around the head (e.g., S-terminalled and penannular hair rings, earrings), neck jewelry (e.g., neck rings, beads), arm jewelry (e.g., bracelets, rings), and dress fittings, as well as weapons (e.g., archery equipment, axes) and implements (e.g., fire-lighting equipment, knives).

Based on the composition of the findings, the cemetery dates back to the period from the first third of the 10th century to the first third of the 11th century. It is believed that the first generation may still have belonged to the conquerors and the last generation may have been buried there during the reign of King (St.) Stephen I. According to a former classification based on the size of the cemetery and the composition of the findings, the cemetery was described as a “commoner cemetery”. In a more recent classification, the cemetery belongs to the 10th century village cemetery group.

The state of preservation of the anthropological material is generally good or medium, excluding the sub-adult skeletons. During the anthropological examinations, 136 adult and 50 sub-adult remains were distinguished, of which 63 were described as male and 83 as female. Based on the distribution of taxonomic features, in addition to the predominance of Europids (88%), a smaller proportion of components belonging to the Mongoloid type were also present (9.3%). Based on biological distance calculations, the population of the cemetery shows similarities to other 10th century and Avar-era series.

#### Appendix A.2.2. Ibrány–Esbóhalom (Szabolcs–Szatmár–Bereg County)

The excavation of the cemetery was carried out between 1985 and 1990 under the leadership of Eszter Istvánovits [13]. The remains of 274 individuals were found in 269 graves. Based on the finds, the 10th century part of the cemetery can be well characterized by the low number of burials with weapon- and horse-riding-related grave goods and dress fittings (e.g., pendant mounts), as well as simple wire jewelry (e.g., bracelets) and implements (e.g., knives, fire-lighting equipment). Within the 10th century part, a group with different ethnicities was also distinguished, based on the grave orientation, burial customs, and findings. In addition to the coins related to the reign of the kings of the Árpád dynasty, the burials of the 11th century were characterized by S-terminalled hair rings, beads, and rings. In virtue of the archeological material, continuity was assumed between the two parts of the site. The use of the cemetery dates back to 940–1075. Based on the number of the graves and the composition of the archeological material, it was characterized as a commoner cemetery. The state of preservation of the anthropological material is medium, often poor. In anthropological studies, 98 men, 82 women, 74 children (inf I–II), and 20 young (juvenile) individuals have been distinguished; thus there is a male surplus of adults. Taxonomic analysis revealed a predominance of Europeans; Mongoloid traits were observed in four individuals. Craniometric analysis showed a discrepancy between the 10th and 11th century parts, raising the question that the two cemetery parts may hide different populations.

#### Appendix A.2.3. Magyarhomorog–Kónyadomb (Hajdú–Bihar County)

The systematic excavation of the cemetery was started by István Dienes between 1961 and 1971 and was finished by László Kovács between 1985 and 1988 [15]. The cemetery has a “pagan” segment with 17 graves from the 10th century characterized by a high number of burials with weapon- and horse-riding-related grave goods and a significant male surplus. The larger part consists of 523 “Christian” graves of the 11–12th centuries in which the gender rate is more balanced. The cemetery is one of the few sites where the burial custom of giving tomb furnishings, which was forbidden by the Christian liturgy, can be observed after the turn of the millennium and the adoption of Christianity (e.g., weapon- and horse-riding-related grave goods in the tombs dated with coins related to the reign of the Árpád dynasty kings). Different types of jewelry were the most common archeological findings: hoops around the head (e.g., S-terminalled hair rings), neck jewelry (e.g., beads), bracelets, and various types of rings. Based on the location of the tombs containing coins, the cemetery started from a central core and expanded evenly toward its edges. During the archeological analysis, the possibility of discontinuity between the two parts of the cemetery arose. Therefore, archeologists suggest that the cemetery separately has a 10th century so-called quarter cemetery and a 11–12th century village cemetery. During the anthropological analysis, 11 men, 3 women, and 3 children of unknown sex were identified in the 10th century part. The state of preservation was generally of medium quality. Based on the taxonomic analysis, the Europid groups dominated (about 60%), but, overall, the proportion of those showing Europo–Mongoloid traits is significant with one individual being classified as Mongoloid type. In paleopathological alterations, developmental abnormalities predominated, which address several further questions about kin relationships within the group. Concerning the 11–12th century village cemetery, the state of preservation of the skeletons was generally low. A total of 126 males, 174 females, 187 adolescents (infantia I–II), and 36 juvenile individuals were distinguished. According to the craniometric analysis, the dolichocranial skulls were dominant, and the taxonomic analysis revealed that skulls with Europid characteristics were in the highest number, but there were also Europo–Mongoloid characteristics and in six cases Mongolid types were present (the state of preservation highly limited the classification). In our analysis, we examined whether the “Christian” part could have been contiguous with the “pagan” part, i.e., it hides descendants of the same population or its population originates from elsewhere. Special attention should also be paid to the possible connections of the previously described cemeteries of Karos and Kenézlő of a similar age.

#### Appendix A.2.4. Nagytarcsa–Homokbánya (Pest County)

In 1967, under the leadership of László Kovács 21 graves were excavated and there are data on another 7 disturbed graves, but the cemetery can only be considered as partially excavated (estimated at about 40–50 graves) [49]. The poor archeological findings consisted of penannular hair rings, twisted neck rings, wire bracelets, rings, and ball buttons. Two burials with weapon-related grave goods (archery equipment, an ax) and four burials with horse-riding-related deposits (e.g., pear-shaped stirrups) were excavated in the cemetery. On the grounds of the findings, the cemetery was dated to the second half of the 10th century. The state of preservation of the anthropological material is moderate, often low. The anthropological analysis [50] identified the skeletons of 8 males, 15 females, and 4 unspecified children. Five skulls belonging to the Europid type proved to be suitable for taxonomic analysis.

#### Appendix A.2.5. Püspökladány–Eperjesvölgy (Hajdú–Bihar County)

The excavation was carried out under the leadership of Ibolya M. Nepper and Márta Sz. Máthé between 1977 and 1982 [11]. A total of 637 graves were found in the cemetery, but due to double burials the remains of 641 individuals were identified. Based on the findings, the cemetery can be divided into two parts. The “pagan” western part (about one-third of the cemetery) dating back to the 10th century is characterized by burials with weapon- (e.g., archery equipment, sabers, swords) and horse-riding-related (pear-shaped stirrups) grave goods, as well as jewelry, such as hoops around the head (e.g., penannular hair rings), necklaces (e.g., beads), and arm/hand jewelry (e.g., bracelets, rings), dress fittings, and implements (e.g., knives, fire-lighting equipment). The other part was likely used after the adoption of Christianity in the 11th century, based on the more common occurrence of coin-dated burials and relatively late grave-good types (e.g., twisted and braided rings, foil beads, S-terminalled hair rings). On the strength of the size of the cemetery and the composition of the archeological material, this cemetery belongs to the commoner cemeteries in the former classification and to the 10–11th century village cemeteries in the more recent classification. The anthropological material is of medium or poor preservation. During the anthropological analysis of the cemetery [11,51], 191 male, 163 female, and 256 sub-adult (unspecified sex) individuals were described. According to studies on craniometric and body height data, continuity was assumed between the 10th century and 11th century parts of the cemetery.

#### Appendix A.2.6. Sárrétudvari–Hízóföld (Hajdú–Bihar County)

The site was excavated between 1980 and 1985 under the leadership of Ibolya M. Nepper [11]. The site, with 262 graves, is considered the largest 10th century cemetery in Hungary. The cemetery contains a very high proportion of burials with weapon- (archery equipment, sabers, axes) and horse-riding-related (e.g., pear-shaped stirrups) grave goods, and the archeological findings consist of jewelry, such as hoops around the head (penannular hair rings), neck jewelry (neck rings, beads), and arm jewelry (e.g., bracelets, beads), dress fittings, and implements (e.g., knives, fire-lighting equipment). Based on the composition of the findings and the lack of coins and grave goods dated to the reign of the kings of the Árpád dynasty, the cemetery can be dated to the 10th century. Formerly it was classified as a commoner cemetery, and in the new classification it belongs to the 10th century village cemeteries.

The skeletal remains are of good/medium preservation. During the extensive anthropological analysis (e.g., [52]), 265 individuals were determined, of whom 98 belonged to sub-adult and 162 to adult categories. Based on the skulls suitable for taxonomic studies, the series shows European characteristics with the presence of Cromagnoid and Nordoid elements.

#### Appendix A.2.7. Szegvár–Oromdűlő (Csongrád County)

The site contained 372 graves which were excavated under the leadership of Gábor Lőrinczy between 1980/1983 and 1996 [53], but many burials (about 75–85) were destroyed due to previous disturbances. Five additional graves were excavated 30–40 m away from the tight array of the cemetery. The archeological material is characterized by jewelry, such as hoop jewelry around the head (penannular hair rings, coiled hair rings, S-terminalled hair rings, hoops with spiral pendants), neck rings, bracelets, and rings, and, less frequently, implements (knives, fire-lighting equipment) and dress fittings (one grave) were excavated. The use of the cemetery dates back to the period between the second third of the 10th century and the middle of the 11th century. Based on its size and the composition of the grave goods, the cemetery was classified formerly as a commoner cemetery of the 10–11th centuries and recently as a 10–11th century village cemetery.

In anthropological studies [54], skeletons of 110 males, 114 females, and 148 sub-adults of indeterminate sex were described. During the taxonomic analysis, the predominance of Europid type skulls (Cromagnoid, Nordoid) was detected in both the 10th and 11th century groups, but a small proportion of Mongoloid features were also described. Based on the craniometric data, it is assumed that a population change occurred at the turn of the 10–11th centuries.

#### Appendix A.2.8. Szegvár–Szőlőkalja (Csongrád County)

The site of 62 graves containing the burials of 63 individuals was excavated in 1979 by Katalin Hegedűs [55]. A burial of opposite orientation (E–W) was found at a distance of 30–35 m from the cemetery array. The poor archeological findings consist of penannular hair rings, beads, wire bracelets, ball buttons, and knives. Horse-riding-related equipment (a fragment of a bridle) and weapons (arrowheads) were unearthed in one burial. Based on the composition of the archeological material, the site dates back to the 10th century, and it was classified formerly as a commoner cemetery and recently as a 10th century villager cemetery.

During the analysis of anthropological findings [55], 25 male and 25 female skeletons were described. Based on taxonomic studies, the population composition is heterogenous; the individuals belonged to the Europid, Mongoloid, and Europo–Mongoloid types.

#### Appendix A.2.9. Vörs–Papkert (Somogy County)

The cemetery was excavated between 1983 and 1993 under the leadership of László Költő, Szilvia Honti, and József Szentpéteri [16]. The cemetery as a whole is still unpublished, but it is dated back to the turn of the 8–9th centuries to the turn of the 10–11th centuries. The 716 excavated burials are mostly from the Late Avar and Carolingian periods, but sporadic burials of 33 people can be dated to the time of the Hungarian conquest. Therefore, the study of the cemetery could help investigate the supposed survival of the Transdanubian Late Avar population through the 9–10th centuries. Extensive anthropological and serological investigations were carried out at the cemetery [56], but contradictory results were obtained (e.g., concerning the sex determination).
